# Digital Cardiovascular Biomarker Responses to Transcutaneous Cervical Vagus Nerve Stimulation: State-Space Modeling, Prediction, and Simulation

**DOI:** 10.2196/20488

**Published:** 2020-09-22

**Authors:** Asim H Gazi, Nil Z Gurel, Kristine L S Richardson, Matthew T Wittbrodt, Amit J Shah, Viola Vaccarino, J Douglas Bremner, Omer T Inan

**Affiliations:** 1 School of Electrical and Computer Engineering Georgia Institute of Technology Atlanta, GA United States; 2 Department of Psychiatry and Behavioral Sciences Emory University School of Medicine Atlanta, GA United States; 3 Department of Epidemiology Rollins School of Public Health Atlanta, GA United States; 4 Department of Medicine Division of Cardiology Emory University School of Medicine Atlanta, GA United States; 5 Atlanta VA Medical Center Emory University Atlanta, GA United States; 6 Department of Radiology Emory University School of Medicine Atlanta, GA United States; 7 Coulter Department of Bioengineering Georgia Institute of Technology Atlanta, GA United States

**Keywords:** vagus nerve stimulation, noninvasive, wearable sensing, digital biomarkers, dynamic models, state space, biomarker, cardiovascular, neuromodulation, bioelectronic medicine

## Abstract

**Background:**

Transcutaneous cervical vagus nerve stimulation (tcVNS) is a promising alternative to implantable stimulation of the vagus nerve. With demonstrated potential in myriad applications, ranging from systemic inflammation reduction to traumatic stress attenuation, closed-loop tcVNS during periods of risk could improve treatment efficacy and reduce ineffective delivery. However, achieving this requires a deeper understanding of biomarker changes over time.

**Objective:**

The aim of the present study was to reveal the dynamics of relevant cardiovascular biomarkers, extracted from wearable sensing modalities, in response to tcVNS.

**Methods:**

Twenty-four human subjects were recruited for a randomized double-blind clinical trial, for whom electrocardiography and photoplethysmography were used to measure heart rate and photoplethysmogram amplitude responses to tcVNS, respectively. Modeling these responses in state-space, we (1) compared the biomarkers in terms of their predictability and active vs sham differentiation, (2) studied the latency between stimulation onset and measurable effects, and (3) visualized the true and model-simulated biomarker responses to tcVNS.

**Results:**

The models accurately predicted future heart rate and photoplethysmogram amplitude values with root mean square errors of approximately one-fifth the standard deviations of the data. Moreover, (1) the photoplethysmogram amplitude showed superior predictability (*P*=.03) and active vs sham separation compared to heart rate; (2) a consistent delay of greater than 5 seconds was found between tcVNS onset and cardiovascular effects; and (3) dynamic characteristics differentiated responses to tcVNS from the sham stimulation.

**Conclusions:**

This work furthers the state of the art by modeling pertinent biomarker responses to tcVNS. Through subsequent analysis, we discovered three key findings with implications related to (1) wearable sensing devices for bioelectronic medicine, (2) the dominant mechanism of action for tcVNS-induced effects on cardiovascular physiology, and (3) the existence of dynamic biomarker signatures that can be leveraged when titrating therapy in closed loop.

**Trial Registration:**

ClinicalTrials.gov NCT02992899; https://clinicaltrials.gov/ct2/show/NCT02992899

**International Registered Report Identifier (IRRID):**

RR2-10.1016/j.brs.2019.08.002

## Introduction

Transcutaneous cervical vagus nerve stimulation (tcVNS) devices have emerged as inexpensive and convenient alternatives to implantable devices for stimulation of the cervical vagus nerve [1]. Over the last half decade, tcVNS-based devices have been approved by the Food and Drug Administration (FDA) for the treatment of migraine and cluster headache, and have demonstrated efficacy in myriad applications, ranging from pain and inflammation reduction to emotional/mental stress attenuation [2-5]. As a noninvasive, nonpharmacologic therapy with minimal side effects [6], the capacity for the widespread use of tcVNS is promising [7]. Yet, to achieve this potential in time-sensitive applications such as in response to traumatic stress, real-time information regarding the patient’s state and response to tcVNS must be incorporated. Thus, a compelling need exists to understand observable biomarker dynamics in relation to tcVNS. This would advance scientific knowledge by uncovering temporal dependencies at finer time resolutions, facilitate real-time predictions of physiological responses to tcVNS for improved treatment titration, and pave the way for optimal estimation approaches to simultaneously track physiological state.

In enabling such closed-loop approaches to tcVNS, initial challenges include (1) identifying noninvasively measured biomarkers of desirable effects, (2) understanding how these biomarkers change dynamically with the delivery of tcVNS for a deepened understanding of the characteristic responses, and, accordingly, (3) predicting each subject’s responses to tcVNS for improved treatment outcomes. In current psychiatric practice, the responses to interventions are primarily assessed through patient reports and qualitative judgments of symptoms [8]. However, these methods are limited by the subjectivity of such reporting and may not be reflective of the true nature of a psychiatric disturbance or subsequent therapeutic response due to factors that include disorder-based perceptual distortions (eg, dissociation), subjective bias, or alternative motivations (eg, malingering) [9]. Alternatively, by studying objective measures that reflect underlying physiological processes, these limitations could potentially be mitigated.

**Figure 1:**
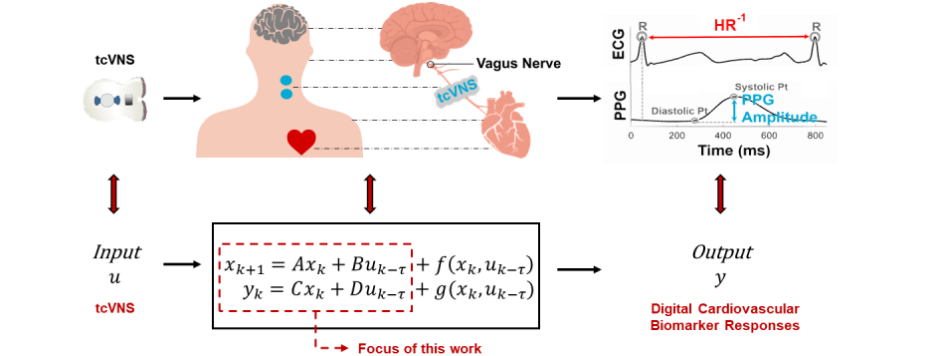
High-level illustration of the modeling task investigated in this study. Transcutaneous cervical vagus nerve stimulation (tcVNS) is treated as the input to the underlying physiological system, while the observed cardiovascular responses found pertinent to tcVNS effects in previous work are treated as the output signals. This work focuses on the dynamics described by linear time-invariant difference equations, formulated as discrete-time state-space equations. The latent state is depicted here as variable x, while f and g are nonlinear functions. HR: heart rate; PPG: photoplethysmogram; ECG: electrocardiogram.

Unfortunately, the existing literature on such objective biomarkers is limited to invasive or obtrusive measures (eg, brain imaging or blood biomarkers) [4,10,11], next-day/longitudinal assessment [2], or static features extracted over minute-long time windows [5,12]. Improving upon this, we applied parametric modeling methods to digital (or physiological) biomarkers [13] that have been deemed to be the most promising in previous work [12]. Specifically, we used state-space models in an input-output formulation to model the dynamic responses of heart rate and photoplethysmogram (PPG) amplitude to tcVNS, as illustrated in Figure 1. State-space models were selected as the machine-learning framework of choice owing to their superior utility in real-time estimation and control applications [14,15]. This work thereby presents a first-of-its-kind dynamic analysis of physiological biomarker responses to tcVNS and furthers the state of the art by: (1) identifying PPG amplitude as a superior digital biomarker to heart rate for the prediction of real-time responses and appraisal of tcVNS effects; (2) quantifying a consistent delay in tcVNS-induced downstream cardiovascular biomarker variation; and (3) uncovering characteristic dynamic response signatures that separate PPG amplitude and heart rate responses to tcVNS from a sham stimulation.

## Methods

### Human Subjects Experiment

As part of a collaborative study approved by the Institutional Review Boards of the Georgia Institute of Technology (#H17126), Emory University School of Medicine (#IRB00091171), SPAWAR Systems Center Pacific, and the Department of Navy Human Research Protection Program, 24 healthy human subjects, including 12 women and 12 men, with a mean age of 31 (SD 9) years, height of 173.4 (SD 8.9) cm, and weight of 77.9 (SD 13.7) kg, were recruited. All subjects had a history of prior psychological trauma, but without current posttraumatic stress disorder or other major psychiatric disorder, and written informed consent was obtained. Conforming to a randomized double-blind protocol spanning 3 days (ClinicalTrials.gov NCT02992899), each subject received four administrations—two on the first day and one on each of the following two days—of either “active” tcVNS or “sham” stimulation. These four administrations were accompanied by no other form of stimulus to focus solely on the effects of tcVNS on human physiology. Overall, 96 doses of either active or sham tcVNS were administered in total to the 24 subjects (with 12 allocated to the active group).

The active and sham devices (gammaCore, electroCore, Basking Ridge, NJ, USA) were identical in both appearance and operation, differing only with respect to the stimulation parameters. The active devices administered voltage signals consisting of five 5-kHz sinusoidal pulses repeating at a rate of 25 Hz, while the sham devices delivered biphasic square pulses at a rate of 0.2 Hz, resulting in a perceptible tingling sensation. For both active and sham devices, electrical stimulation was delivered transcutaneously to the left side of the neck, targeting the cervical vagus nerve projection. At specified times, the researcher initiated the device and adjusted the stimulation amplitude (ranging from 0 to 5 arbitrary units [AU], corresponding to 0-30 V and 0-14 V for the active and sham device, respectively) to as high as the subject could comfortably endure (active: 3.0 [SD 0.8] AU; sham: 4.4 [SD 1.2] AU). The amplitude was then kept fixed for the remainder of a 120-second period, following which the device automatically stopped, reducing its stimulation amplitude to zero. This 2-minute timeframe replicates the programmed administrations onboard the tcVNS devices that are currently in use [16]. For further protocol and stimulation details, the reader is referred to Gurel et al [5].

### Physiological Sensing, Signal Processing, and Biomarker Extraction

**Figure 2:**
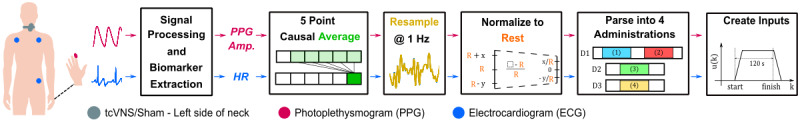
Physiological sensing, signal processing, and modeling preparation. Twenty-four subjects (12 active) underwent a clinical protocol, wherein the electrocardiogram (ECG) was measured with electrodes placed in a three-lead configuration and the photoplethysmogram (PPG) was measured from the fingertip in a transmissive LED-photodiode setup. Transcutaneous cervical vagus nerve stimulation (tcVNS) or sham stimulation was administered at predefined times, where the exact stimulation location was identified by locating the left carotid pulse. Following signal processing and biomarker extraction, the biomarkers were prepared for modeling via 5-point causal averaging, resampling to 1 Hz, normalizing to rest, and finally parsing into 4 separate vectors associated with the 4 tcVNS/sham administrations studied. By referencing stimulation initiation, the corresponding input amplitude waveforms were then constructed to replicate device administration. Amp.: amplitude; D1: day 1; D2: day 2; D3: day 3.

Electrocardiogram (ECG) and transmissive PPG signals, taken from the finger, were continuously measured at the locations displayed in Figure 2 using the Biopac RSPEC-R and Biopac PPGED-R systems (Biopac Systems, Goleta, CA, USA), respectively. All data were acquired at a 2-kHz sampling rate using the Biopac MP150 16-bit data acquisition system.

To extract the instantaneous heart rate from the ECG signal, finite impulse response bandpass filtering (passband of 0.6-40 Hz) was applied to cancel out-of-band noise while maintaining the waveform shape [17]. By then detecting the R-peaks, the instantaneous heart rate was computed in beats per minute by taking the reciprocal of the time length, in minutes, between each pair of R-peaks (R-R interval). These R-peaks were subsequently leveraged to beat-separate the bandpass-filtered PPG signals (passband of 0.4-8 Hz [18]). PPG amplitude (in AU), was then calculated on a beat-by-beat basis by subtracting the global minimum of each PPG beat from its global maximum.

The focus on heart rate and PPG amplitude as the biomarkers of interest for this work was based on a rationale that both cardiac and vascular downstream responses to stimulus were of interest scientifically and may indicate different autonomically mediated mechanisms following tcVNS. Heart rate is a hallmark measure of the cardiac response to changes in autonomic tone and is controlled by both the sympathetic and parasympathetic branches of the autonomic nervous system. Parasympathetic decreases in heart rate are mediated by the release of acetylcholine that binds to muscarinic receptors in the heart, whereas sympathetic increases in heart rate are mediated by the release of epinephrine and norepinephrine that bind to beta-1 receptors in the heart. PPG is a measure of peripheral blood volume pulse, and represents a surrogate measure of vasodilation or vasoconstriction resulting primarily from changes in sympathetic tone. Peripheral vasoconstriction is mediated by alpha-1 receptors in the smooth muscle of the vasculature [19].

### Preparing the Biomarker Time Series for Modeling

Following feature extraction, the heart rate and PPG amplitude values existed as beat-to-beat time series of nonuniform sampling rates (due to variability in heart rate). Thus, prior to any modeling, a few time-series processing steps were employed (Figure 2). First, each subject’s biomarker time series were prepared using a causal moving average of 5 data points (approximately 5-second, rectangular windows) to attenuate high-frequency artifacts. The filtered time series were then resampled at a constant frequency of 1 Hz to satisfy uniform sampling rate requirements [20], followed by normalization to each day’s resting value (subtracting and then dividing by rest) to account for intersubject variability during our subsequent population-level analysis.

To then separately investigate the effects of tcVNS/sham administration on each of the two biomarkers, the resultant time series were parsed according to the 4 administrations per subject. Based on the data available, parsing was achieved by leaving 60 seconds of data prior to each 120-second administration and retaining 120 seconds of data postadministration, for a total of 300 seconds per administration. Note that missing data at the end of the 300-second interval relevant to our analysis affected 3 administrations among the 96 collected, and therefore the corresponding data vectors were shortened accordingly.

The accompanying input data were then created for each of the 192 subject-administration-biomarker combinations (2 biomarkers × 4 administrations × 24 subjects). This was accomplished by modeling the relative tcVNS/sham amplitude delivered to each subject with pulse-like trapezoidal signals that replicated the ramping and stabilization of true stimulation. These inputs were formed by passing a boxcar input of 120-second width and unit amplitude through a 5-point moving average filter. Since stimulation amplitude remains the only variable modulated during tcVNS/sham administration, stimulation amplitude was specified as the input variable, as done in related work [21,22]. This ensured that the subsequently analyzed input-induced effects modeled the tcVNS-induced effects under study. Note that the digital biomarker response dynamics modeled in this study correspond to a particular therapy—FDA-approved tcVNS—that exhibits equivalent input bandwidths across all administrations. Therefore, modeling the input-output relationship for the specific input variability exhibited during practical device usage remains invaluable to future analysis and development. For further reasoning and evidence behind this approach, please see Multimedia Appendix 1.

### State-Space Modeling and Cross-Validation

Considering the established diversity in VNS outcomes, which itself remains an active area of research [23], subject-specific models were developed for our study, leaving the identification of consistencies across relevant subject groupings for the subsequent model analysis phase. To address biomarker differences, heart rate and PPG amplitude were modeled separately to foster comparison between the estimated systems and responses for each of the two biomarkers. This maneuver to disentangle outputs that seemingly respond to tcVNS/sham simultaneously derives from a result in state-space input-output modeling, where the absence of output feedback–controlled effects on the input under study allows for the disentangling of multiple-output systems [24] (see Multimedia Appendix 1).

The forthcoming equations and corresponding explanations will thus depict single-input single-output systems. The discrete-time state-space model structure in innovations form is governed by the following two difference equations:

*x_k+1_*=*Ax*_k_ +*Bu_k–τ_* + *Ke_k_*   **(1)**

*y_k_*=*Cx_k_* + *Du_k–τ_* + *e_k_*   **(2)**

where *k* represents the discrete time step, *τ* represents the input delay of the system (also known as the “dead time” between changes in input and resultant changes in system behavior), *y* ∈ **R** represents the output, *u* ∈ **R** represents the input, *x* ∈ **R**^M^ represents the latent state of the dynamical system (referred to as the state vector), where *M* is referred to as the model order of the system, *e* ∈ **R** is the residual computed as the innovation estimate, and *A* ∈ **R**^M×M^, *B* ∈ **R**^M×1^, *C* ∈ **R**^1×M^, *D* ∈ **R**^M×M^, and *K* ∈ **R**^M×1^ are matrices consisting of free parameters that are estimated to best describe the data provided. In the context of this work, the scalar *D* is set to zero, as it quantifies the feedthrough component’s contribution on the output (ie, the static contribution of the input that circumvents dynamics entirely).

**Figure 3:**
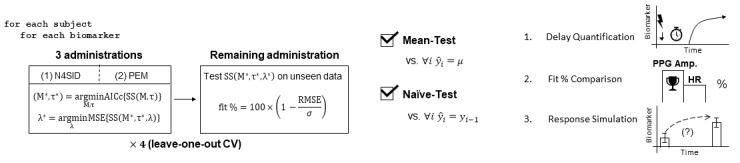
Modeling, optimization, and cross-validation; quality assurance; and analysis. (Left) Modeling, optimization, and testing were performed in a leave-one-out cross-validation (CV) scheme, where, out of the four administrations, each administration was considered once as the unseen test set. The model order (M) and input delay (τ) were optimized by minimizing the small sample size-corrected Akaike information criterion (AICc). For each model order-input delay combination, a state-space model was trained by first initializing the parameter estimates using subspace estimation (N4SID); this was then followed by prediction error minimization (PEM) to refine the parameter estimates. Ridge regression was then performed for this specific model configuration by iterating lambda (λ) logarithmically over a specified interval and minimizing the mean square error. The 1-step-ahead prediction performance was evaluated using a fit percentage formula based on the root mean square error (RMSE) normalized by the standard deviation of the data (σ). (Middle) For quality assurance beyond subjective satisfaction in visual results, the models were evaluated against two objective baseline tests from the literature: the mean test and the naïve test. (Right) To extract the model information pertinent to a deepened dynamic understanding of biomarker responses, (1) optimal input delays were compiled to assess the expected response latency following stimulation onset, (2) the biomarkers were compared against each other to identify superiority in monitoring dynamic changes following transcutaneous cervical vagus nerve stimulation (tcVNS), and (3) the responses were quantifiably visualized via controlled simulation to produce population trajectories of expected dynamic changes following transcutaneous cervical vagus nerve stimulation (tcVNS) administration, in comparison to sham.

In this study, we initialized model estimates using subspace identification [25] and then employed prediction error minimization to ameliorate any limitations of the computationally cheaper subspace method [26]. In accordance with our subject-specific modeling objective, leave-one-out cross-validation was used to train and evaluate the state-space models. Specifically, each of the 4 administrations was used for testing exactly once, with the remaining 3 administrations used for model estimation. This eventually resulted in an overall 4 models per biomarker per subject, following the optimization process detailed below. Figure 3 illustrates this cross-validation process.

### Regularization and Hyperparameter Optimization

The small sample size-corrected Akaike information criterion (AICc) was employed to select the optimal model configuration (M*, τ*); the AICc was used instead of the standard AIC due to the ratio between training data points, *N,* and the number of parameters, *p,* in the largest candidate model remaining less than 40 [27,28]. For technical details on usage, please refer to Multimedia Appendix 1.

To optimize τ, based on a previous effort to subjectively annotate the delays between tcVNS and biomarker changes [29], the interval was set to 0 ≤τ≤35; this was to circumscribe a 99% confidence interval about the reported result of 18 seconds (SD 7). For *M*, the lack of broadband input constrained the parameter total to below the order of input persistence of excitation for any candidate model [30]. The order of persistent excitation is estimated by counting the number of distinct frequencies with spectral content larger than a set threshold [31]. By performing this computation iteratively across all input signals used in this study, including those associated with datasets missing data points, the order never decreased below 50. Thus, our optimization interval was safely restricted to a maximum of 50 parameters, corresponding to model order 10 for modal form estimates (see Multimedia Appendix 1).

Once the optimal state-space model was selected for each training set of 3 administrations, the model was then regularized separately via ridge regression [32]. The parameter λ was selected by minimizing the mean square error over the interval λ ∊ [10^–15^, 10^4^]. The lower bound was selected due to MATLAB’s machine epsilon for double-precision floating points (2^–52^) and the upper bound was selected during an experimentation period. These optimal, regularized model estimates were then used for the remaining analyses. The methods described in this subsection and the previous section correspond to the box on the left in Figure 3.

### Fit Percentage Definition and Baseline Testing

The models were evaluated against two established baselines for dynamic modeling tasks: the mean test and the naïve test. The mean test involves comparing out-of-sample 1-step prediction performance vs mean predictors, and the naïve test involves comparing 1-step prediction performance vs the naïve predictor [33-35] (see Multimedia Appendix 1 for further details). This out-of-sample testing is depicted in the box on the right of Figure 3 (left). Figure 3 (middle) also summarizes the baseline testing.

The metric used herein for evaluation is the fit %, defined as:

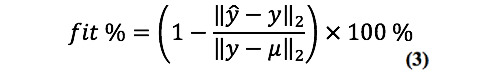



where *ŷ*=[*ŷ*_1_ *ŷ*_2_… *ŷ*_N_]*^T^* represents the predicted output values from time step 1 to N and *y*=[*y*_1_*y*_2_… *y*_N_]*^T^* represents the true output. This exact metric has been widely used to quantify time-series model validity (eg, [21,24]), along with its variants (eg, [33]). Note that the above fit % formula can be equivalently rewritten as (1 – RMSE/σ) × 100%, where RMSE is the root mean square error between the predicted and true values and σ is the standard deviation of the data. Thus, the fit % used here is commonly referred to as a fit % metric based on standard deviation – normalized RMSE, which represents an estimate of the output variability the model can accurately reproduce [24]. This is seen to be the case when comparing the fit % to the coefficient of determination, given by:

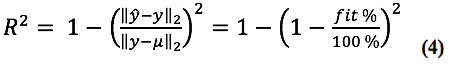



demonstrating that the fit % simply replaces the mean square error and variance with the RMSE and standard deviation, respectively. It thereby exhibits improved spread for RMSE < σ/2.

### Model Configuration and Fit Comparisons

Figure 3 (right) illustrates the subsequent methods of analysis. To determine the presence of any notable differences in predictability between the biomarkers in question, the biomarker fit percentages were compared. Additionally, the optimal model orders associated with each biomarker’s models were compared to determine whether any model complexity differences could be posited. In this case, unlike fit %, lower model orders are generally favored, as they signify relatively simpler systems.

To further investigate the optimal model configurations, the automatically optimized input delays, τ*, for each of the two biomarkers were compiled. These two sets of latencies were then compared against the manually annotated delays of previous work. As detailed in Gurel et al [29], this manual labeling was performed independently by three investigators with an interannotator agreement of 90%.

### Statistical Testing

With 4 fit % values, model orders, and input delays obtained per subject-biomarker combination (corresponding to the 4 models produced via cross-validation), all quantities were first averaged across all 4 models prior to comparison/compilation. To examine biomarker differences in fit % and model order, pairwise *t* tests or Wilcoxon signed-rank tests were employed for normally and nonnormally (tested using the Shapiro-Wilk test) distributed variables, respectively. For comparison of the delay results, after failing to reject normality and sphericity (tested using the Mauchly sphericity test), a one-way repeated-measures analysis of variance was used; α=.05 was used as the level of significance for these comparisons.

### Investigating Biomarker Responses to tcVNS vs Sham

As a final analysis step, we investigated the tcVNS-induced response dynamics captured through modeling by simulating both the active and sham models forward from a controlled state. To leverage the previously resampled biomarker time series, we assembled a second set of plots corresponding to the true experimental responses. To facilitate qualitative comparisons, both sets were constructed by compiling/simulating the true/artificial biomarker time series such that 10 seconds existed prior to the true/simulated stimulus administration and 120 seconds remained afterward. The 120 seconds corresponds to the 2-minute poststimulus period used during modeling, and the 10 seconds were included to better understand the true biomarker values prior to stimulation.

#### Resampled Experimental Responses

For each of the 4 administrations for the 24 subjects, the heart rate and PPG amplitude time series were extracted by simply considering the values produced as a result of the modeling preparation steps. With resampled and normalized time series in hand, each biomarker’s overall response was constructed by first averaging the biomarker responses across all 4 administrations, followed by additional average and SEM calculations across all 12 subjects in each device group.

#### Simulated Model Responses

To visualize the biomarker dynamics captured by the models, each model was simulated forward by (i) setting the initial conditions to zero (ie, initializing the system at its equilibrium) to guarantee equivalence between active and sham time series (setting *x*_1_=0 guarantees that the system will remain there for as long as no stimulus is present); (ii) constructing an input waveform equivalent to the input used during the modeling process, nonzero between time points 10 and 130 seconds; and (iii) solving the difference equations forward in time for each model, ignoring the contribution of *Ke* and *e* in Equations (1) and (2), respectively, as these quantities represent the unmodeled aspect of the system, along with noise, lumped into residual terms [36]. Each biomarker’s overall response was constructed by averaging across all 4 simulated model responses, followed by calculation of the average and SEM across each device group. For further details regarding the advantages of this approach and insight into the subsequent simulation analysis, please refer to Multimedia Appendix 1.

## Results

### Baseline Test Results

**Figure 4:**
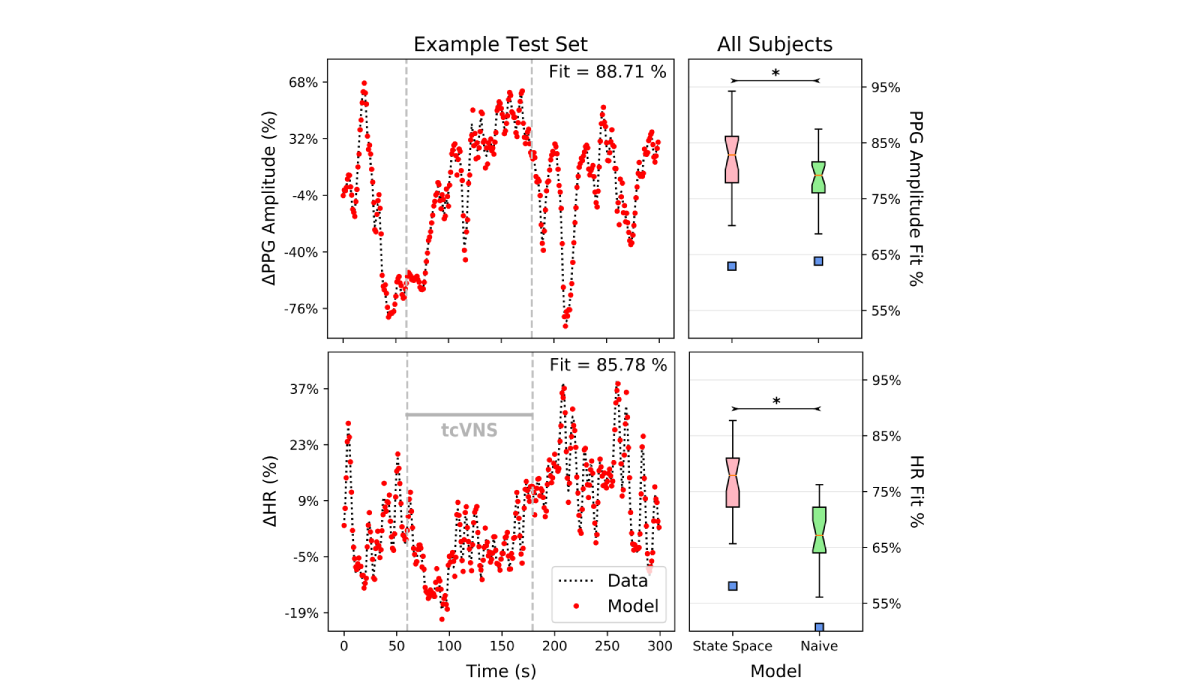
Example model predictions vs true data, along with naïve test box plot results for the entire sample. (Left) Both model vs data plots shown originate from one administration of a single active transcutaneous cervical vagus nerve stimulation (tcVNS) subject. Each biomarker’s model was trained on the three other tcVNS administration datasets available for this subject; the test results on the remaining unseen dataset in a 1-step-ahead prediction task are shown. The gray dashed lines demarcate the time frame in which tcVNS was administered, and the fit percentages are calculated as previously defined. The y-axis represents relative changes from rest in percent form. (Right) The regularized, optimal state space models strongly satisfied both the naïve test and the mean test. The statistically significant (*P*<.001) naïve test results are shown, where the box plots indicate results for the entire sample; * denotes statistical significance. The blue squares indicate outlier points, where outliers lie above the upper quartile or below the lower quartile by more than 1.5 times the interquartile range. Significance (*P*<.001) held for the mean test as well for both biomarkers (not shown).

Figure 4 (left) displays the heart rate and PPG amplitude data in response to a single administration of active tcVNS from a representative subject. For illustrative purposes, the corresponding model predictions are overlaid. To produce these predictions for each biomarker, the model was trained on the remaining three datasets for this subject and tested on this particular dataset.

The naïve test results across all subjects are shown on the right side of Figure 4. Note that the mean predictors, by definition, always produce fits of 0%. Hence, the models for both biomarkers passed both the naïve test and the mean test, exhibiting significantly higher fit percentages than the naïve predictors (*P*<.001) and mean predictors (*P*<.001).

### Modeling Amenability Comparison

Table 1 summarizes the results from the biomarker fit % and model order contrast. The fit of each of the PPG amplitude models was significantly better than that of the heart rate models (*P*=.03), albeit without compensation through an increase in model order/complexity. Model order did not differ between heart rate and PPG (*P*=.14).

**Table 1:** Mean (SD) fit % and model orders for each biomarker’s state-space model.

Metric	Heart rate	PPG^a^ amplitude
Fit %	76.67 (6.96)	81.64 (7.07)
Model order	9.52 (0.38)	9.39 (0.34)

^a^PPG: photoplethysmogram.

### Automatically Estimated Input Delays vs Manually Labeled Onset Delays

**Figure 5:**
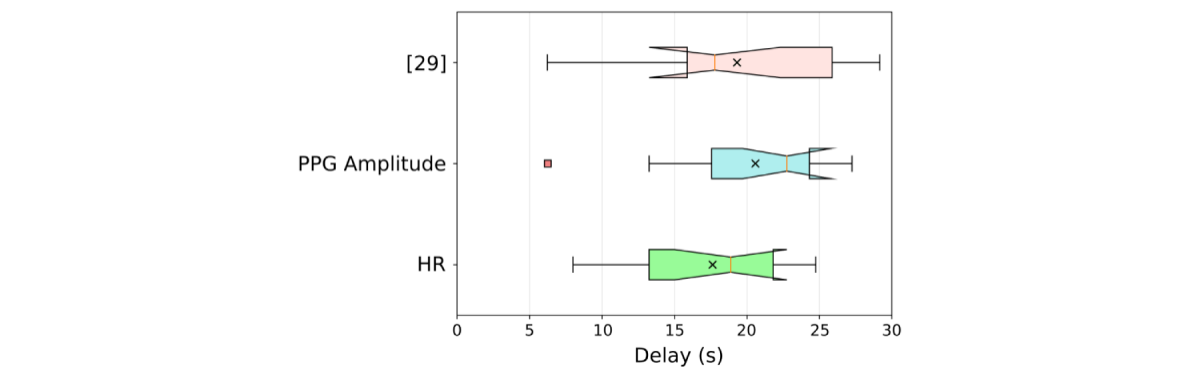
Delayed effects of transcutaneous cervical vagus nerve stimulation (tcVNS) in downstream cardiovascular biomarkers. By including input delay as a free parameter necessitating optimization, the objective, quantitative state-space model optimization process described in this paper reproduced the tcVNS delay findings of the manual, subjective annotations of [29]; no statistically significant differences existed between the three sets of onset delays. The coral-colored square represents an outlier, and the black crosses represent the means.

Figure 5 shows the automatically estimated input delays for both biomarker models, as well as the manually annotated result from previous work [29]. The mean input delay for the heart rate and PPG amplitude state-space models was 17.65 seconds (SD 5.17) and 20.58 seconds (SD 5.81), respectively, and the mean manually labeled delay was 18 seconds (SD 7). No statistically significant differences were found between the three sets of onset delays (F_2, 22_=0.71, *P*=.51, *η*^2^_p_=.06).

### Biomarker Responses to tcVNS vs Sham

**Figure 6:**
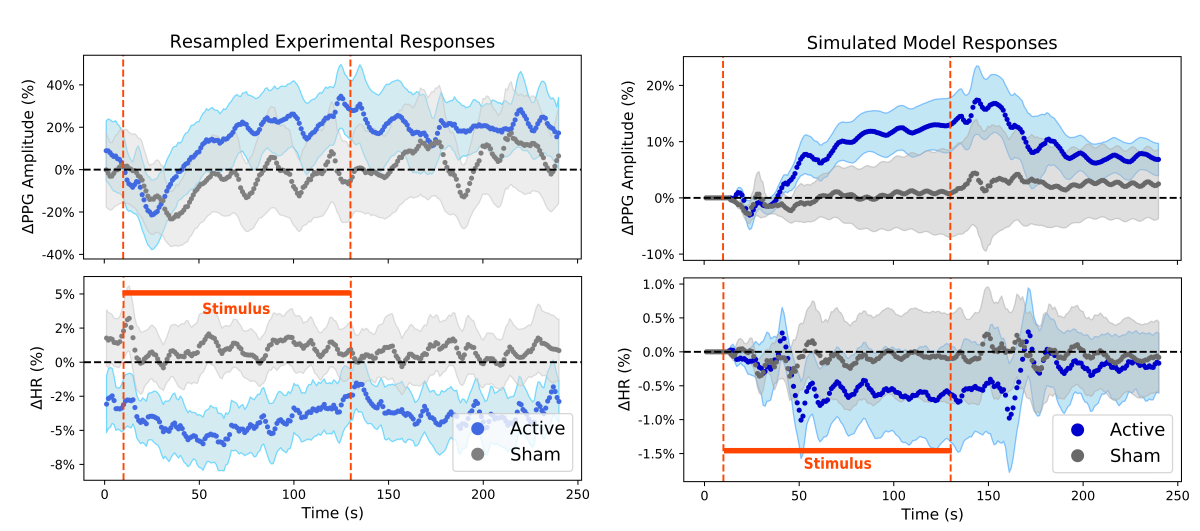
Dynamic responses to transcutaneous cervical vagus nerve stimulation (tcVNS) for heart rate (HR) and photoplethysmogram (PPG) amplitude. The curves themselves, along with their accompanying shaded regions, represent average (SEM). (Left) True responses, resampled for population averaging and subsequent dynamic modeling. The y-axis values represent relative changes from rest in percent form, and the orange dashed lines demarcate the period of active/sham stimulus administration (t ∈ (10, 130) seconds). (Right) Simulated responses to tcVNS, produced by solving the state-space model difference equations forward in time. The y-axis values represent relative changes from rest in percent form, and the orange dashed lines demarcate the period of simulated active/sham stimulus administration (t ∈ (10, 130) seconds).

Figure 6 shows the heart rate and PPG amplitude responses to tcVNS or sham administration. The graphs on the left correspond to the resampled experimental responses, and those on the right correspond to the simulated model responses. Although conclusions are weakened when comparing the true active and sham responses due to the lack of controlled initial conditions, one can posit possible dynamic response signatures for PPG amplitude and heart rate in response to active tcVNS when comparing against their respective sham counterparts. Through modeling and simulation, we remedy these initial condition concerns. Rubin causality is therefore exhibited in response to tcVNS, allowing for causal inference to be legitimately made by comparing the active and sham responses. Moreover, the underlying dynamic response signatures of tcVNS can be better visualized in comparison to the uneventful sham responses.

## Discussion

### PPG Amplitude is a More Reliable and Predictable Biomarker of tcVNS Effects

Based on the results in Table 1, we found that the models were able to reproduce the PPG amplitude data significantly better than the heart rate data, albeit without requiring any increase in model complexity to compensate. PPG amplitude may thus be a more trackable dynamic marker of the physiological responsiveness to tcVNS than those previously investigated. Notably, such biomarkers have thus far remained elusive in the existing tVNS literature [37]. Hence, in conjunction with the consistency of PPG amplitude discovered in previous work [5,12], the finding that PPG amplitude remains more amenable to our modeling approach suggests that PPG amplitude may serve as a superior biomarker for real-time tcVNS systems, and thus should be given precedence in a multimodal sensing setting.

Biomarker characteristics that could explain this distinction involve measurement location and physiological origin. Heart rate is a cardiac measure originating centrally from the heart, whereas the PPG signals measured in this study were sensed at the periphery—in particular, transmissively at the fingertip. As described in the Methods section, a clear physiological distinction exists; these differences may in fact explain the relative contrasts observed in this study, as well as the previous difficulties encountered in identifying a trustworthy biomarker derived from the heart (eg, heart rate variability measures) [37].

Cardiovascular biomarkers extracted from peripheral processes, mediated solely by the sympathetic nervous system (eg, vasoconstrictor sympathetic nerve activity [38] and PPG amplitude [18]), have shown repeated success in capturing the effects of tVNS. An explanation for this may involve physiological control. In comparison to the peripheral blood vessels and their sympathetically mediated vasoconstriction, heart rate is regulated by both the sympathetic and parasympathetic nervous systems; additionally, the heart, owing to its critical role in maintaining blood supply to the entire body, is subject to far tighter regulation [19,39]. The peripheral vasculature thus remains more susceptible to external modulation, or in a systems sense, external “disturbances.” Thus, a promising approach for sense-and-react systems that seek to assess the effects of external modulation may be to leverage peripheral biomarkers that measure quantities subject to looser homeostatic control.

### tcVNS-Induced Effects are Delayed in Digital Cardiovascular Biomarkers

Figure 5 illustrates the consistency found between the optimized input delays of this work and the manually annotated latencies of prior work [29]. Note, however, that these delay values of approximately 15 seconds may in fact be overestimates in certain situations due to the approximate 5-second ramp-up period during administration. Since this aspect was accounted for during modeling, one can reasonably deduct 5 seconds from the delays listed for purposes involving closed-loop system design and hypothesizing the dominant mechanism of action. Our reasoning is that for a closed-loop system adapted for a particular subject, the threshold of comfort will likely be known, thereby eliminating the ramp-up period. To provide quantitative evidence for a dominant mechanism of action, protocol-related delays are not necessarily relevant to determining the underlying pathways that tcVNS affects when causing downstream cardiovascular effects. Nevertheless, as determined by the notched confidence intervals shown in Figure 5, mechanistically relevant delays remain at latencies of >5 seconds, even after factoring in the 5-second deduction. Thus, for closed-loop tcVNS systems, a delay greater than 5 seconds must be taken into consideration and designed for accordingly [40,41].

In providing quantitative evidence for the likely dominant mechanism of action for tcVNS effects on downstream physiology, we here highlight the two prevalent hypotheses for tcVNS at either the cervical or auricular branch of the vagus nerve [42,43] (see Multimedia Appendix 1 for a detailed illustration). The first hypothesis involves electrical activation of vagal efferents terminating at the heart [44]; this mechanism would in fact induce the expected decrease in heart rate, which has been frequently cited in previous animal studies (eg, [45]) and was also found to be the case here. However, this hypothesis may not necessarily explain the sympathetically mediated effects observed at the periphery during tVNS administration (eg, vasoconstrictor sympathetic nerve activity [38]). The second hypothesis implicates afferent vagal stimulation as a pathway to brain activity in autonomically relevant brain areas, followed by downstream effects induced by resultant efferent signaling. Although brain imaging and electrophysiological measurements have confirmed the activation of vagal afferents during tcVNS [10,46], it remains unclear whether these “bottom-up” effects serve as the dominant cause for the resultant autonomic efferent activity.

The results presented herein seem to align with the latter hypothesis: namely, that the delayed effects modeled—and subjectively observed in previous work—are a byproduct of afferent vagal activity, processing in the brain, and resultant efferent-mediated autonomic effects. Synthesizing previous sensing and measurement studies of tcVNS, afferent signatures (P1-N1 vagal somatosensory evoked potentials) tend to occur within 1 second of amplitude stabilization [46]. Considering that finite element modeling results suggest that only A and B vagal fibers can be stimulated through gammaCore tcVNS [47], and that efferent B fiber conduction velocities range from 5 to 10 m/s [44], we would expect that if the dominant pathway for cardiac effects occurred through direct efferent stimulation, these effects would occur within 2-3 seconds of amplitude stabilization. Instead, we conclude that the dominant tcVNS effects on cardiovascular physiology likely occur after 5-10 seconds have elapsed, following amplitude stabilization (after reasonably deducting a 5-second ramp-up period from the ~15-second delays reported). This suggests that downstream cardiovascular effects are likely mediated by the prolonged afferent-brain-efferent mechanism of action.

### Distinct Dynamic Signatures Characterize tcVNS-Induced Effects

Heart rate and PPG amplitude responses were visualized both for resampled data averaged across administrations and subjects, and for simulated responses produced by solving the estimated difference equations forward in time. As shown in Figure 6 (left), the average active PPG amplitude response to tcVNS clearly exceeded the average sham response to tcVNS, although they coincidentally initialized at similar relative values. In contrast, the average active heart rate graph displays a transient decrease in response to tcVNS that lasts about 50 seconds, followed by a return to prestimulus values. Interestingly, this agrees with recent findings in the auricular tVNS literature, which noted 3-4% transient drops in heart rate that recovered over the course of 30 seconds following stimulation onset [48]. Unlike the PPG amplitude responses, the average heart rate responses of the two device groups initialized with an almost 4% relative difference between them, which is an important point to consider when comparing the two trajectories. Although the average active heart rate response seems starkly different from the average sham heart rate response, if one were to artificially shift the two responses’ initial conditions vertically to level the playing field, the active response would remain beneath the sham response, but definitively by less. Moreover, this disregards the concerns associated with performing such an artificial transformation for causal inference and comparison [49].

This issue of Rubin causality (see Multimedia Appendix 1) is ultimately resolved on the right side of Figure 6. Since the dynamic models developed thus far were simulated forward from the same equilibrium condition, we arrived at population-level characteristic responses to tcVNS that exhibited the necessary equivalent prestimulus behavior. With this added causal inference power, we not only observed apparent similarities between the plots on the left and right of Figure 6, but we also discovered evident differences between the active and sham groups in their responses to tcVNS. In particular, the active group’s simulated PPG amplitude responses significantly exceeded the relatively flat sham PPG amplitude trajectories; analogously, but in the opposite direction, the active heart rate trajectories displayed a clear decrease in comparison to the sham responses, where the sham responses again remained relatively constant. Thus, the exact same modeling and simulation methodology were applied to data from both device groups, and yet stark differences arose in the characteristic responses to stimulation. Furthermore, the sham group displayed relatively subdued responses to the simulated stimulus, as expected. These results serve as further validation for the current approach and ultimately imply the presence of distinctive dynamic signatures that characterize the continuous-time effects of tcVNS on digital cardiovascular biomarkers.

### Limitations and Future Work

A few limitations are to be noted for this study. Although nonlinear approaches to difference equation modeling are generally discouraged at the outset unless expert knowledge or sufficient evidence seems to suggest otherwise [24], nonlinear dynamics exist in general. Nevertheless, in agreement with previous findings demonstrating that a considerable percentage of dynamic variability exhibited by cardiovascular biomarkers such as heart rate can be modeled linearly [50], we found that approximately 80% of the biomarker variability observed can still be predicted accurately. Hence, this paper presents a “best linear approximation” that could further be built upon in future work directed at characterizing the nonlinearities of tcVNS responses [24].

Although all tcVNS clinical protocols reported to date have used the gammaCore device to which the present results readily apply, future control approaches will need to venture beyond this standardized waveform and vary parameters during stimulation to achieve desired regulation goals, while simultaneously maintaining user safety. This would additionally help in satisfying the stringent input requirements for system identification [24], which are conditions that have not been met thus far.

Finally, we note that biomarkers other than PPG amplitude and heart rate were found to be statically relevant in quantifying tcVNS effects in previous work [5], although static PPG amplitude and heart features were found to be the most salient [12]. Thus, a multimodal closed-loop system that utilizes signals other than ECG and PPG may benefit from further application of such dynamic modeling and analysis.

### Conclusions

In this work, we studied heart rate and PPG amplitude responses to tcVNS and derived three key findings by approaching this question from a dynamic modeling perspective. First, PPG amplitude demonstrates preeminence in both modeling amenability and active vs sham response separation, suggesting its superiority as a digital biomarker for real-time response prediction and tcVNS effect quantification. Second, a consistent delay of greater than 5 seconds exists between tcVNS onset and downstream cardiovascular biomarker responses. Latency must therefore be considered and accounted for appropriately during clinical monitoring and closed-loop system design. Moreover, this delay may provide measurable evidence for the dominance of the hypothesized vagal afferent-to-brain-to-autonomic efferent pathway in downstream cardiovascular modulation. Lastly, state-space models can successfully predict heart rate and PPG amplitude dynamics in response to tcVNS, and can help to identify the characteristic dynamic signatures that separate these biomarker responses to tcVNS from sham stimulation. This dynamic modeling and analysis thereby deepens our understanding of tcVNS effects and lays the groundwork for future closed-loop approaches in time-sensitive applications.

## Appendix

Multimedia Appendix 1 Supplementary material.

## Abbreviations

AICc: small sample size-corrected Akaike information criterion
AU: arbitrary units
ECG: electrocardiogram
FDA: Food and Drug Administration
PPG: photoplethysmogram
RMSE: root mean square error
tcVNS: transcutaneous cervical vagus nerve stimulation
