# Supplementary material for: Digital Cardiovascular Biomarker Responses to Transcutaneous Cervical Vagus Nerve Stimulation: State-Space Modeling, Prediction, and Simulation
Source: JMIR Mhealth Uhealth. 2020 Sep 22;8(9):e20488. doi: 10.2196/20488 (PMC7539162; doi:10.2196/20488)
Supplement: Multimedia Appendix 1 [file mhealth_v8i9e20488_app1.pdf]

Appendix

Pulse Input Response Modeling

The trapezoidal pulse-input assumption is applied scrupulously in our work, as the lack of broadband input restricts model applicability to only those of inputs exhibiting similar frequency content [1]. Additionally, only parametric modeling in the time-domain remains legitimate, provided that the number of parameters estimated does not exceed input excitation [2]. In contexts such as classical system identification, for example, where the goal of model-fitting involves learning the impulse response/transfer function of the underlying system to effectively predict the output for any given input, these obstacles can preclude progress [3]. In contrast, when it comes to modeling response dynamics following a particular therapy – in our case, FDA-approved tcVNS – that remains consistent across all administrations, modeling the input-output relationship for the specific input variability exhibited during practical device usage remains invaluable to future analysis and development.

Notably, this response modeling approach parallels interrupted time series analysis (ITSA), a cross-disciplinary statistical method applied in fields such as public health [4], [5] and socioeconomics [6], [7] for quasi-experimental assessment of an intervention's effects. A particular brand of methods utilizes time series modeling approaches (e.g., autoregressive integrated moving average with exogenous input (ARIMAX) models) and formulate the intervention problem similarly, where the intervention variable, I_t, can be modeled analogously to the input signal used in this work [8]. The advantages we highlight here and leverage in analysis are the unequivocal causality implications afforded through subsequent model simulation and comparison.

As a final note on rationale, as elucidated in [9] and stated explicitly in terms of frequency spectrum in [10], modeling should be undertaken with application in mind [11]. We therefore argue that considering the pre-programmed nature of the tcVNS devices in use today, understanding their specific dynamic effects on human physiology remains a necessary step toward developing closed-loop tcVNS systems of the future [12]–[14]. Note here that this fixed-waveform tcVNS assumption is in no way a new deliberation, as prior work has also restricted modeling and analysis to the stimulation waveform delivered by the gammaCore tcVNS device [15]–[18]. And even in the transcutaneous auricular VNS (taVNS) case where alternate stimulation parameters (frequency and time length) have been explored [19], the input parameters vary during administration as they do here, i.e., the frequency of stimulation is kept fixed during the administration period, and the amplitude is modulated as we model here, where the device is turned on, the amplitude is kept fixed, and then the device is turned off at the conclusion of a fixed time period. Thus, our approach remains largely applicable to a broad array of tVNS researchers, and our results hold immediate significance in that they apply to the frequency and administration time length used by tcVNS devices today.

SIMO Systems as Multiple SISO Systems

Let S represent a single-input multiple-output (SIMO) system given by the following two state-space equations:

$$x_{S_{k+1}} = f\left(x_{S_k}, u_k\right)$$
$$y_{S_k} = g\left(x_{S_k}, u_k\right)$$

where $x_S \in \mathbb{R}^n, u \in \mathbb{R}, y_S \in \mathbb{R}^m$. Assume that u is not a function of y_S.
The output equation, $y_{S_k} = g\left(x_{S_k}, u_k\right)$, can be equivalently written as m separate scalar output equations, i.e., $\forall_{i \leq m} \;\; y_{S_{k_i}} = g_i(x_{S_k}, u_k)$, where i denotes the i-th component of the vector equation. Thus, the dynamics described by S can be equivalently expressed as m single-input single-output (SISO) systems $\left\{S_j\right\}_{j=1}^{m}$, where S_j is given by

$$x_{S_{k+1}} = f\left(x_{S_k}, u_k\right)$$
$$y_{S_{j_k}} = g_j\left(x_{S_k}, u_k\right)$$

Thus, by replicating the state dynamics, $x_{S_{k+1}} = f(x_{S_k}, u_k)$, for each of the m systems and considering each component of $g(\cdot)$ as its own output equation, a SIMO system can be expressed as multiple SISO systems. ■

Note that a key assumption made in the proof above is that u is not a function of y_S. In practice, this can be assessed by inspecting the cross correlations between u_i and y_j for $i > j$.

State-Space Parameters in Modal Form

Model order, M, and input delay, τ, represent model configuration variables (i.e., hyperparameters) that dictate structural aspects of the two equations, and thus, should be specified prior to final model estimation. Model order also dictates the number of free parameters necessitating estimation. Since state space models are not unique in representation (i.e., different A, B, C can represent the same system) we narrowed our parameter search to A matrices in modal form [20], leaving all elements in B, C, and K free.

Modal form entails a block diagonal matrix where each block on the diagonal corresponds to an eigenvalue or pair of eigenvalues of the system (if complex eigenvalues exist, these blocks correspond to complex conjugate pairs). For example, if a state space system was of order 4 with two distinct real eigenvalues λ_1, λ_2 and a pair of complex conjugate eigenvalues, $\lambda_{3,4} = \sigma \pm j\omega$, A would be of the following form:

$$A = \begin{bmatrix} \lambda_1 & 0 & 0 & 0 \\ 0 & \lambda_2 & 0 & 0 \\ 0 & 0 & \sigma & -\omega \\ 0 & 0 & \omega & \sigma \end{bmatrix}$$

To arrive at the total number of state space parameters, notice that $A \in \mathbb{R}^{M\times M}$. For even-ordered matrices, the number of parameters to estimate is $2M$, corresponding to two parameters per row of A. This is due to the fact that even if an eigenvalue is estimated as purely real, the imaginary component had to remain free during this estimation to come to this conclusion. For odd-ordered matrices, one of the eigenvalues *must* be real, and therefore, the number of parameters in A to estimate remains $2M - 1$ (the imaginary component corresponding to the real eigenvalue is no longer a free parameter). Since the systems estimated in this paper were of SISO form, B, C, and K were all of dimension M, and thus, $p \leq 2M + M + M + M = 5M$, where p is the number of free parameters for state-space estimation.

Optimizing Model Configuration using the AICc

The AICc can be computed for our model estimation purposes as follows:

$$AICc = N\,log\left(\frac{1}{N}\sum_{k=1}^{N}\epsilon^2(k|\hat{\theta})\right) + 2p + N(log(2\pi)+1) + \frac{2p(p+1)}{N-p-1}$$

where $\hat{\theta} \in \mathbb{R}^p$ represents the estimated parameters, and $\epsilon(k|\hat{\theta})$ is the prediction error at timestep k given the parameter estimate $\hat{\theta}$. Minimizing the AICc coincides with an attempt to optimize the trade-off between the in-sample mean square error $\frac{1}{N}\sum_k \epsilon^2(k|\hat{\theta})$ and model complexity, quantified by p. Thus, this method of minimizing the AICc over all possible configurations can be summarized by the relation

$$\underset{M\leq 10\,,\ \ \tau\leq 35}{argmin}\ \ AICc(\{SS\ (M,\tau)\}) \xrightarrow{yields} SS_{selected}(M^*,\tau^*)$$

where $SS_{selected}(M^*,\tau^*)$ corresponds to the selected state-space configuration, and $\{SS(M,\tau)\}$ represents the set of all possible state-space configurations.

Baseline Testing

Mean-predictors are defined for each dataset as a predictor obeying the following discrete-time output equation:

$$\forall k \quad \hat{y}_k = \mu \triangleq \frac{1}{N}\sum_{i=1}^{N} y_i$$

where $\hat{y}_k$ represents the predicted output at timestep k, y_i represents the true output datapoint at timestep i, and μ is defined as the mean output value. The naïve-predictor is defined as:

$$\hat{y}_1 = y_1, \;\; \forall k > 1 \;\; \hat{y}_k = y_{k-1}$$

Notice that, by definition of the fit % included in the paper's main text, the mean-predictor will always return 0%. Models with fit % less than 0% are hence usually considered inoperative, in the sense that the true dynamics of the output were likely left uncaptured, as guessing the mean at each time step would have produced better performance. Similarly, the naïve-test evaluates a separate null hypothesis that involves predicting that the next output value will remain equivalent to the present measurement; in particular, the naïve-test investigates whether one should reject the hypothesis that the time series could be better modeled as a discrete-time martingale (stochastic process where $\mathbb{E}\left[y_k \middle| \{y_i\}_{i=1}^{k-1}\right] = y_{k-1}$ [21]). If one fails to reject this hypothesis, then the models can be considered unsuccessful in gleaning relevant dynamical information, and the modeling approach should be revisited. These simplistic approaches should not be downplayed, however, as results from practical forecasting applications have repeatedly demonstrated that mean and naïve-predictors often outperform models of additional complexity [22].

Further Details on Simulated Model Response Analysis

Although residual terms improve the modeling and prediction process by considering inherent process noise, measurement noise, and ever-present unmodeled aspects of the data [1], when homing in on the input-output dependency itself, these terms are to be ignored, as we are more interested in how the biomarkers respond following the specific input under investigation. Hence, an advantage of this approach to response modeling and simulation is the dynamic disentanglement it offers, facilitating concentrated analysis on the specific contributions of the modeled input u.

Notice that the same exact modeling process was applied to datasets from both active and sham subjects; thus, any differences identified within these plots hold value, as contrasting responses would suggest the presence of dynamic response signatures, consistent enough to quantify on a population level, that characterize the biomarker responses to tcVNS. By imposing Rubin causality through the control of initial conditions for both groups prior to simulated stimulation administration, thereby guaranteeing the equivalence of the "control time series" and the "treatment time series" prior to stimulus [8], stronger conclusions can be made regarding the modeled cause and effect between stimulus and response by analyzing the resultant biomarker trajectories. This contrasts what the resampled experimental responses afford, as the uncontrolled initial conditions leave less to be reliably learned from the data, bar evident trends and timing information.

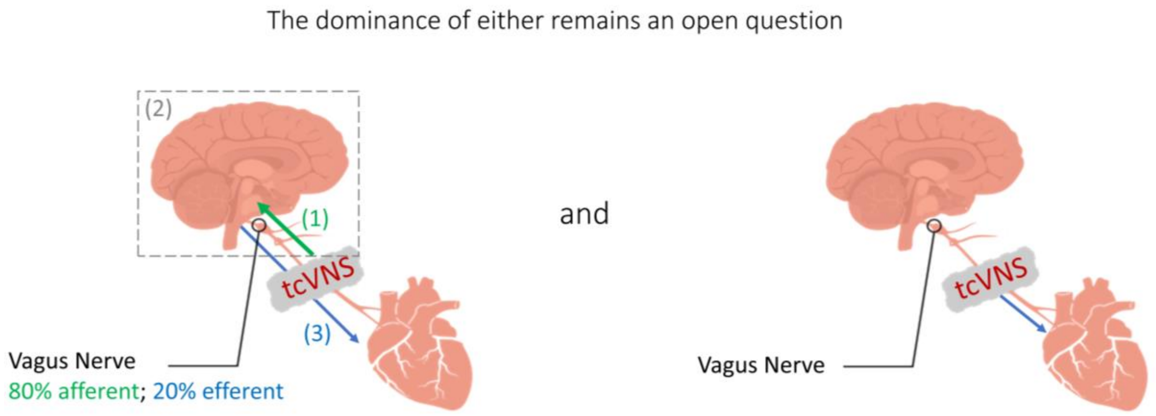

Appendix figure 1. Illustration of the two prevalent hypotheses for the dominant mechanism action underlying tcVNS effects on downstream physiology.

Illustration of Two Prevalent Hypotheses for tcVNS Mechanism of Action

See Appendix figure 1, along with the explanation included in the main text.

Monte-Carlo Validation of Delay Optimization Approach

To investigate whether the consistency in reported delays could in fact be an artifact of the processing and optimization process, we conducted a Monte-Carlo validation experiment. Iterating over a set of specified model orders and input delays, known models were constructed through random sampling of the A, B, C, and K matrices, replicating our processing and model optimization process thereafter. Specifically, the model orders, M, iterated over were 2, 4, 6, 8, and 10 (model order was iterated over to experiment with processes of varying complexity); and the input delays, τ, experimented with were 1, 5, 10, 15, 20, 25, 30, and 35. Five models were constructed for each combination of model order and input delay, specifying A by uniformly sampling M discrete-time eigenvalues from the unit circle (ensuring compliance, of course, with complex conjugates), while B, C, and K were determined by sampling each of the vector elements from the standard normal distribution.

These models were then simulated forward four times, setting u to be the trapezoidal pulse input used during modeling and e to be Gaussian white noise. The resultant output data were then prepared for modeling as done for the HR and PPG amplitude time series in the main text (see figure 2). Modeling this input-output data as in the tcVNS-biomarker case (see figure 3), we observe a reassuring relationship between estimated and true input delay (see violin plots of Appendix figure 2). In particular, for $\tau \in [1, 25]$, the estimated delay distributions center themselves around the true delay; moreover, a noticeable positive trend exists between true delay and the median of the estimated delays. Based on this result, we find it justifiable to believe that the true latency (protocol delays included; see Discussion) we seek to estimate of tcVNS effects on HR and PPG amplitude falls somewhere within the interval $\tau \in [15, 25]$, as the box plots reported in the main

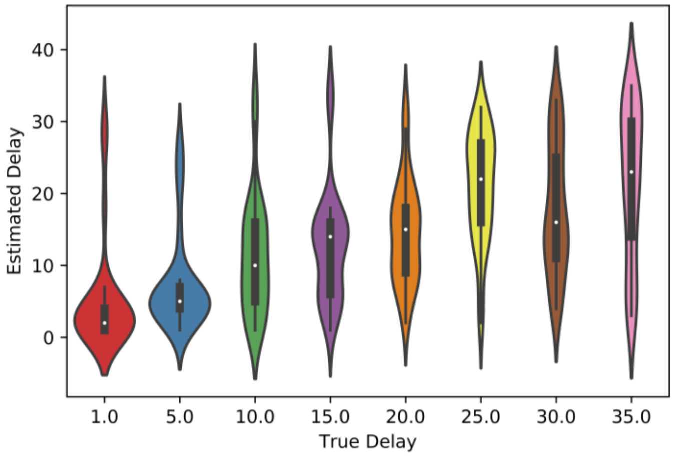

Appendix figure 2. Delay optimization results for the Monte-Carlo validation experiment. The violin plots shown each contain 25 datapoints, corresponding to the 25 random models of varying complexity constructed for each input delay. The colored regions that surround the thinner box plots in black (with medians shown in white) correspond to kernel density estimates. The y-axis corresponds to optimal delay values, while the x-axis represents true delay values.

text (see figure 5) visualize distributions that lie somewhere within this true delay interval, according to Appendix figure 2.

Data and Code Sharing Statement

The HR and PPG amplitude time series that support the findings of this study are available upon reasonable request from the authors. The time series preparation, state-space modeling, and subsequent analysis code is made publicly available at: https://github.com/asimgazi24/tcVNS_Response_Modeling

Appendix References

[1] L. Ljung, *System Identification: Theory for the User*, Second Edi. Prentice Hall, 1999.

[2] R. Mukkamala and R. J. Cohen, “A forward model-based analysis of cardiovascular system identification methods,” 2000.

[3] T. Chen, H. Ohlsson, and L. Ljung, “On the estimation of transfer functions, regularizations and Gaussian processes-Revisited,” *Automatica*, vol. 48, no. 8, pp. 1525–1535, Aug. 2012.

[4] J. Lopez Bernal, S. Cummins, and A. Gasparrini, “Interrupted time series regression for the evaluation of public health interventions: a tutorial,” *Int. J. Epidemiol.*, pp. 348–355, 2017.

[5] J. B. Albu *et al.*, “An interrupted time series analysis to determine the effect of an electronic health record-based intervention on appropriate screening for type 2 diabetes in urban primary care clinics in New York city,” *Diabetes Care*, vol. 40, no. 8, pp. 1058–1064, Aug. 2017.

[6] E. Kontopantelis, T. Doran, D. A. Springate, I. Buchan, and D. Reeves,

"Regression based quasi-experimental approach when randomisation is not an option: Interrupted time series analysis," *BMJ*, vol. 350, Jun. 2015.
[7] C. Bonham, E. Fujii, E. Im, and J. Mak, "The Impact of the Hotel Room Tax: An Interrupted Time Series Approach," 1992.
[8] D. McDowall, R. McCleary, E. Meidinger, and R. Hay, *Interrupted Time Series Analysis*. SAGE Publications, Inc., 2011.
[9] L. Ljung and T. Glad, *Modeling of Dynamic Systems*, First Edit. Pearson, 1994.
[10] J. Schoukens and L. Ljung, "Nonlinear System Identification: A User-Oriented Road Map," *IEEE Control Syst.*, vol. 39, no. 6, pp. 28–99, Dec. 2019.
[11] H. H. Hjalmarsson, "System Identification of Complex and Structured Systems," *Eur. J. Control*, pp. 3–4, 2009.
[12] N. Z. Gurel *et al.*, "Toward closed-loop transcutaneous vagus nerve stimulation using peripheral cardiovascular physiological biomarkers: A proof-of-concept study," in *2018 IEEE 15th International Conference on Wearable and Implantable Body Sensor Networks, BSN 2018*, 2018, vol. 2018-Janua, no. April, pp. 78–81.
[13] N. Z. Gurel *et al.*, "Quantifying Acute Physiological Biomarkers of Transcutaneous Cervical Vagal Nerve Stimulation in the Context of Psychological Stress," *Brain Stimul.*, vol. 13, no. August, pp. 47–59, 2019.
[14] P. D. Ganzer and G. Sharma, "Opportunities and challenges for developing closed-loop bioelectronic medicines," *Neural Regeneration Research*, vol. 14, no. 1. Wolters Kluwer Medknow Publications, pp. 46–50, Jan-2019.
[15] C. Brock *et al.*, "Transcutaneous cervical vagal nerve stimulation modulates cardiac vagal tone and tumor necrosis factor-alpha," *Neurogastroenterol. Motil.*, vol. 29, no. 5, p. e12999, May 2017.
[16] E. Frangos and B. R. Komisaruk, "Access to Vagal Projections via Cutaneous Electrical Stimulation of the Neck: fMRI Evidence in Healthy Humans," *Brain Stimul.*, vol. 10, no. 1, pp. 19–27, Jan. 2017.
[17] R. Nonis *et al.*, "Evidence of activation of vagal afferents by non-invasive vagus nerve stimulation: An electrophysiological study in healthy volunteers," *Cephalalgia*, vol. 37, no. 13, pp. 1285–1293, Nov. 2017.
[18] A. P. Mourdoukoutas, D. Q. Truong, D. K. Adair, B. J. Simon, and M. Bikson, "High-Resolution Multi-Scale Computational Model for Non-Invasive Cervical Vagus Nerve Stimulation," *Neuromodulation*, vol. 21, no. 3, pp. 261–268, 2018.
[19] B. W. Badran *et al.*, "Short trains of transcutaneous auricular vagus nerve stimulation (taVNS) have parameter-specific effects on heart rate," *Brain Stimul.*, vol. 11, no. 4, pp. 699–708, Jul. 2018.
[20] P. A. Ioannou and J. Sun, *Robust adaptive control*, First Edit. Dover Publications, 2012.
[21] H. Stark, J. W. (John W. Woods, and H. Stark, *Probability, statistics, and random processes for engineers*. Pearson, 2012.
[22] J. S. Armstrong, *Principles of forecasting : a handbook for researchers and practitioners*. Kluwer Academic, 2001.
